# Real‐World Outcomes of Thromboprophylaxis With Apixaban in Patients With Newly Diagnosed Multiple Myeloma, the MyelMeXa Study

**DOI:** 10.1002/jha2.70325

**Published:** 2026-06-08

**Authors:** K. Alexandra Sanchez, Gabriela Cesarman‐Maus, Michelle Flores‐Cruz, Beatriz Cabello, Ramiro Espinoza, Omar Eduardo Fernandez‐Vargas

**Affiliations:** ^1^ Department of Hematology Instituto Nacional de Cancerología Mexico City Mexico

**Keywords:** Anti‐Xa oral anticoagulants, multiple myeloma, thromboprophylaxis, thrombotic risk scores, venous thromboembolism

## Abstract

**Background:**

Multiple myeloma (MM) carries a high venous thromboembolism (VTE) risk; aspirin shows limited efficacy, while factor‐Xa inhibitors show promise.

**Objectives:**

Evaluate apixaban for primary thromboprophylaxis in newly diagnosed MM (NDMM).

**Methods:**

Prospective single‐center study of consecutive patients receiving apixaban, followed for VTE and bleeding.

**Results:**

Eighty‐seven patients (median follow‐up of 11 months) were included; no major bleeding, deep vein thrombosis, or pulmonary embolism occurred; two developed symptomatic superficial vein thrombosis.

**Conclusions:**

Apixaban was safe and associated with very low rates of clinically significant thrombotic and bleeding events, supporting further evaluation for thromboprophylaxis in NDMM.

**Trial Registration:**

The authors have confirmed clinical trial registration is not needed for this submission.

## 1 Introduction

Venous thromboembolism (VTE), which includes proximal deep venous thrombosis (DVT) and pulmonary embolism (PE), is a frequent complication in multiple myeloma (MM), particularly during early treatment. Its pathogenesis is multifactorial, driven mainly by prothrombotic therapies including immunomodulatory drugs (IMiDs), high‐dose dexamethasone (> 160 mg/month), and anthracyclines [1]. Additional contributors include reduced mobility and patient‐related factors like comorbidities, body mass index, and race.

The cumulative incidence of VTE exceeds 10% and remains clinically relevant despite modern regimens [1, 2]. While proteasome inhibitors—particularly bortezomib—may reduce thrombotic risk [3].

Current guidelines recommend routine thromboprophylaxis for all patients with newly diagnosed MM (NDMM) in the absence of contraindications [1, 4]. Historically, aspirin (ASA) has been the most commonly used strategy because of its low cost, ease of administration, and early randomized data suggesting comparable efficacy to low‐molecular‐weight heparins (LMWH) and vitamin K antagonists (VKA). However, real‐world data show relevant VTE rates (6.4%–16.1%) and non‐negligible bleeding risk (∼4%–5%) [5, 6].

In our retrospective study of NDMM patients with predominantly ASA prophylaxis, VTE occurred in 8% within 6 months, with major bleeding (MB) and clinically relevant non‐major bleeding (CRNMB) rates of 4.8% and 4.4%, respectively [5]. Most patients were classified as intermediate‐to‐high thrombotic risk by myeloma‐specific thrombotic risk scores (TRS) (IMPEDE‐VTE, PRISM, and SAVED), highlighting the need for improved strategies.

Oral factor‐Xa inhibitors have emerged as promising alternatives, with low VTE rates and favorable bleeding profile compared with ASA [6, 7, 8]. In comparative analyses, oral factor‐Xa inhibitors have shown the lowest VTE rates among prophylactic strategies [2].

While treatment patterns in MM have been described in Latin America (LATAM) [9], prospective data evaluating modern thromboprophylaxis strategies–particularly oral anti‐factor Xa inhibitors–remain limited. We therefore conducted the MyelMeXa study, a prospective real‐world cohort evaluating apixaban for primary thromboprophylaxis in patients with NDMM, with the aim of assessing the effectiveness and safety of apixaban and evaluating the performance of myeloma‐specific TRS.

## 2 Methods

### 2.1 Study Design and Setting

This prospective, single‐center observational cohort study was conducted at the National Cancer Institute (Mexico). Consecutive NDMM patients were enrolled between November 2023 and December 2025. Following our prior study, a standardized protocol was implemented whereby all patients with NDMM were followed in parallel from diagnosis by both the Myeloma Clinic and the Cancer and Thrombosis Clinic (CTC), with evaluations at the CTC every 4–6 weeks to monitor bleeding and thrombotic events. TRS assessment was documented at baseline when the chemotherapy regimen was initially prescribed.

### 2.2 Participants

Consecutive adults (≥ 18 years) with NDMM defined by IMWG criteria were included. Exclusion criteria were contraindications to anticoagulation or need for anticoagulant/antiplatelet therapy for other indications. No patients met the exclusion criteria.

### 2.3 Outcomes

Primary endpoints were image‐confirmed VTE, MB, and CRNMB. Secondary endpoints included TRS performance, which was planned to be explored using ROC curves.

VTE diagnosis required objective confirmation by Doppler ultrasonography or computed tomography (CT). Assessment of thrombosis was based on clinical suspicion, as routine screening for asymptomatic thrombosis was not performed. Cardiac or peripheral arterial thrombotic events were to be confirmed by Doppler ultrasonography, magnetic resonance imaging, or CT angiography, supplemented by ECG and cardiac biomarkers. Bleeding was classified using ISTH criteria [10]. Thrombotic risk was assessed using IMPEDE‐VTE and SAVED scores in all patients, and PRISM in those with available metaphase cytogenetics. All patients completed at least 6 months of follow‐up; however, only 37 patients reached 12 months of follow‐up at the time of analysis.

### 2.4 Statistical Analysis

Baseline characteristics and outcomes were summarized descriptively. Cumulative incidence of thrombosis was estimated using competing‐risk methods (death as a competing event). Overall survival (OS) was measured from diagnosis.

## 3 Results and Discussion

### 3.1 Patient Population and Treatment

Eighty‐seven patients were included (36.8% women; mean age 57.8 ± 9.4 years). At diagnosis, 43.7% had ECOG ≥ 2 and 46.0% had ISS Stage III disease. IgG myeloma was present in 40.2%, IgA in 23.0%, and light‐chain‐only disease in 35.6%. Bone disease was present in 87.4%, hypercalcemia in 18.4%, renal failure in 26.4%, and anemia in 37.9%, as per IMWG criteria (Table 1).

**TABLE 1:** Baseline demographic and disease characteristics of 87 patients with NDMM.

Variable	Value
Age in years, mean ± SD	57.8 ± 9.37
Sex, *n* (%)	
Female	32 (36.8)
Male	55 (63.2)
ECOG performance status, *n* (%)	
< 2	49 (56.3)
≥ 2	38 (43.7)
BMI category, *n* (%)	
Normal weight	37 (42.5)
Overweight	35 (40.2)
Obesity	15 (17.2)
ISS, *n* (%)	
I	22 (25.3)
II	24 (27.6)
III	41 (47.1)
ISS‐R, *n* (%)	
I	13 (14.9)
II	45 (51.7)
III	28 (32.2)
Not available	1 (1.2)
Bone disease, *n* (%)	
Lytic lesions	70 (80.5)
Pathological fractures	6 (6.9)
None	11 (12.6)
Hypercalcemia, *n* (%)	16 (18.4)
Renal failure, *n* (%)	23 (26.4)
Anemia, *n* (%)	33 (37.9)

Abbreviations: BMI, body mass index; ECOG, Eastern Cooperative Oncology Group; ISS, International Staging System; R‐ISS, Revised ISS.

Most patients received high‐dose dexamethasone (88.5%) and IMiDs (85.1%), with proteasome inhibitors in 68.9% (bortezomib 48.3%, carfilzomib 20.7%), and daratumumab in 12.6%. No erythropoiesis‐stimulating agents were used. Agent posology followed conventional dosing schedules, with no changes in lines of therapy during the first 6 months; however, while high‐dose dexamethasone was common, dose variations in routine practice were not systematically documented.

### 3.2 Thrombotic Risk Stratification

Most patients were at high risk by IMPEDE‐VTE (75.9%) and SAVED (88.5%), with moderate agreement between scores (*κ* = 0.58). PRISM (available in 75 patients) was categorized mostly as intermediate risk (94.6%). Risk assignment was largely driven by IMiD use and high‐dose dexamethasone exposure (Table 2).

**TABLE 2:** Distribution of individual thrombosis risk score components.

Thrombotic risk score	Risk factor^a^	Points assigned	Patients with a risk factor, *n* (%)
IMPEDE‐VTE	IMiDs	+4	74 (85.1)
	BMI > 25 kg/m^2^	+1	50 (57.5)
	HD‐Dex (> 160 mg/month)	+4	77 (88.5)
	LD‐Dex (< 160 mg/month)	+2	10 (11.5)
SAVED	HD‐Dex (> 160 mg/month)	+2	77 (88.5)
	LD‐Dex (< 160 mg/month)	+1	10 (11.5)
PRISM	IMiD exposure	+3	71 (94.7)
	High‐risk cytogenetics	+2	21 (28.0)

^a^ Risk score components not shown had no occurrences in the study cohort.

### 3.3 Thromboprophylaxis

All patients received prophylactic dose of apixaban (2.5 mg twice daily) except two, who initially received rivaroxaban. Thromboprophylaxis was planned for at least 12 months, with duration and adherence guided by treating physicians, and long‐term prophylaxis was allowed. Temporary interruptions occurred in selected cases (e.g., procedures) and were managed following the CHEST 2022 guidelines. Two patients required modification of anticoagulants during follow‐up, one due to renal dysfunction (switch to unfractionated heparin), another due to access (switch to rivaroxaban).

### 3.4 Thrombotic Events

No DVT, PE, or arterial thrombosis occurred during follow‐up. Two patients (2.2%) developed symptomatic superficial venous thrombosis. Both events occurred in high‐risk patients (according to IMPEDE‐VTE and SAVED) and were associated with additional risk factors: immobility in one case, and temporary interruption of anticoagulation during a procedure in the other. The 12‐month cumulative incidence of thrombosis was 2.5% (95% CI 0.46–7.81).

### 3.5 Bleeding Outcomes

No MB occurred and only two patients (2.2%) experienced CRNMB (epistaxis and two episodes of abnormal vaginal bleeding), supporting a favorable safety profile compared to published rates of combined major and CRNMB with ASA of ∼4%–5% [5, 6].

### 3.6 Survival

At a median follow‐up of 11 months (IQR 5.4–17.6), 10 deaths occurred, primarily due to myeloma progression (*n* = 7). Twelve‐month OS was 88.4%.

### 3.7 Clinical Implications

Guidelines recommend thromboprophylaxis in NDMM [1, 11], with increasing support for oral anti‐factor‐Xa inhibitors in higher‐risk patients [4], including a meta‐analysis suggesting better efficacy and safety compared to ASA [12]. However, myeloma‐specific TRS have limited predictive accuracy, with frequent VTE events occurring even in low‐risk groups [6]. This raises the question of whether a uniform prophylactic strategy may be appropriate in NDMM, irrespective of risk categorization.

In this cohort, despite predominantly intermediate‐to‐high thrombotic risk, no clinically significant VTE events (DVT/PE), minimal bleeding, and only 2.2% of symptomatic superficial vein thrombosis were observed in patients receiving apixaban. Though our study does not directly compare prophylactic strategies, findings appear favorable compared to VTE rates reported for ASA (6.4%–16.1%), LMWH (1.2%–5%), and warfarin (4–8.2) [5, 6, 13]. Furthermore, our results align with a prior retrospective study that evaluated prophylactic apixaban in 70 patients with NDMM, with no VTE events occurring within the first 6 months [14].

Regarding safety, apixaban demonstrated low bleeding rates, with CRNMB occurring in two patients (2.2%) and no MB events. These findings also compare favorably with ASA cohorts [5, 6].

With respect to thrombotic risk stratification using myeloma‐specific TRS, the low number of events precluded meaningful evaluation of score performance. The study was therefore underpowered to evaluate these scores; however, the low event rate observed with apixaban prophylaxis suggests that differences in baseline risk may be less clinically relevant.

### 3.8 Strengths and Limitations

Limitations include the single‐center design, potential referral bias, lack of systematic screening for asymptomatic thrombosis, and limited long‐term follow‐up. The predominance of intermediate‐ and high‐risk patients limits definitive conclusions regarding truly low‐risk individuals. Treatment exposure was not modeled as a time‐varying variable; consequently, variations in dexamethasone dosing in routine clinical practice were not systematically captured. Only one patient had dose de‐escalation following autologous hematopoietic cell transplant within the first 6 months of therapy.

Strengths include the prospective design and close clinical follow‐up in a relatively homogeneous NDMM population. As apixaban was the primary anticoagulant used, results cannot be extrapolated to other oral anti‐Xa inhibitors. Emerging data from the prospective COBRRA trial show that apixaban is associated with a lower bleeding risk than rivaroxaban when used at therapeutic doses; however, it remains unclear whether this difference persists at prophylactic dosing [15]. While retrospective data suggest acceptable outcomes with rivaroxaban 10 mg daily as primary prophylaxis for MM [6], these findings require confirmation.

Overall, our data support further prospective evaluation of oral anti‐Xa inhibitors as a promising alternative thromboprophylaxis strategy in NDMM, potentially irrespective of baseline TRS‐defined risk categories.

## 4 Conclusions

Apixaban thromboprophylaxis in patients with NDMM was well tolerated and associated with very low rates of clinically significant thrombotic and bleeding events. Most patients were classified as having an intermediate‐to‐high thrombotic risk, mainly driven by treatment‐related factors. The predictive performance of scores could not be assessed due to the low event rate. These findings support further prospective evaluation of apixaban as primary thromboprophylaxis in NDMM.

## Author Contributions

K.A.S., G.C.M., and O.E.F.V. conceptualized and designed the study. K.A.S., M.F.C., B.C., and R.E. collected and curated the data. K.A.S. and O.E.F.V. performed the statistical analysis. K.A.S, G.C.M, and O.E.F.V. drafted the manuscript. All authors contributed to the interpretation of the data, critically revised the manuscript, and approved the final version for publication.

## Funding

The authors have nothing to report.

## Ethics Statement

The study protocol was reviewed and approved by the Institutional Ethics and Research Committees of the Instituto Nacional de Cancerología, Mexico City, Mexico (approval numbers 023/055/HEI and CEI/059/23). The study was conducted in accordance with the principles of the Declaration of Helsinki. All participants provided written informed consent prior to enrollment in the study.

## Conflicts of Interest

The authors declare no conflicts of interest.

## Data Availability

The data supporting the findings of this study are available from the corresponding author upon reasonable request. Due to institutional and ethical restrictions related to patient confidentiality, the data are not publicly available.
